# Cerebral and bone multidrug resistant tuberculosis treated with the BPaLM regimen: case report

**DOI:** 10.3389/fphar.2026.1796484

**Published:** 2026-05-12

**Authors:** Marina Calamelli, Caterina Davoli, Roberta Gerami, Maurizio Gulletta, Sofia Lovatti, Alessandro Pavesi, Benedetta Rossi, Alberto Matteelli

**Affiliations:** 1 Department of Clinical and Experimental Sciences, Clinic of Infectious and Tropical Diseases, ASST Spedali Civili Hospital, University of Brescia, Brescia, Italy; 2 Clinic of Infectious and Tropical Diseases, ASST Spedali Civili, Brescia, Italy; 3 Department of Experimental Medicine and Public Health, Clinic of Infectious and Tropical Diseases, School of Advanced Studies, University of Camerino, Camerino, Italy

**Keywords:** Bones, Central Nervous System, Multidrug resistance, Treatment, Tuberculosis

## Abstract

**Introduction:**

The BPaLM regimen is recommended for the treatment of pulmonary and extrapulmonary multidrug resistant tuberculosis, with the exception of cerebral and bone localization due to uncertainties on adequate drug concentrations in these tissues.

**Methods:**

We report two MDR-TB cases with cerebral and bone disease treated with BPaLM at the Clinic of Infectious and Tropical Diseases in Brescia, Italy. Clinical data were collected through a comprehensive review of the electronic medical records, diagnostic results and patient interviews. The patients provided written informed consent for the publication of clinical data in anonymous form.

**Results:**

Patient 1, affected by disseminated TB involving lungs, lymph nodes and bones, completed 7 months of therapy, six of which with the BPaLM regimen, and demonstrated no evidence of relapse at 1-year follow-up. Patient 2, presenting with disseminated TB including cerebral and bone localisation, received the BPaLM regimen that was discontinued after 4 months due to adverse events.

**Discussion:**

Our report contributes to the limited clinical evidence on the use of BPaLM in cerebral and bone RR/MDR-TB. Of the two patients treated with this regimen, one did not complete therapy; however, treatment interruption was attributable to adverse events rather than lack of efficacy. Further evidence is urgently required on shorter regimens such as BPaLM for the treatment of extrapulmonary tuberculosis involving the brain and bones.

## Introduction

Drug-resistant tuberculosis (DR-TB) remains a major global health challenge, with profound implications for patients, communities, and healthcare systems (World Health Organization, 2025a). The World Health Organization (WHO) estimates that approximately 390,000 new cases of multidrug-resistant or rifampicin-resistant tuberculosis (RR/MDR-TB) occurred in 2024, resulting in around 150,000 deaths (World Health Organization, 2025b). In 2022, the WHO endorsed the first 6-month regimen for the treatment of RR/MDR-TB, composed of bedaquiline, pretomanid, linezolid, and moxifloxacin (BPaLM) (World Health Organization, 2022). This regimen, however, was not indicated for central nervous system (CNS), bone, and miliary TB, mainly due to unavailability of data on the penetration of bedaquiline and pretomanid in these tissues (World Health Organization, 2022). To date, only a single case report has documented the use of BPaL in CNS TB (Howell et al., 2021), and no published data exist on its use in bone TB.

Here, we describe two MDR-TB cases with cerebral or bone localization, treated off-label with the BPaLM regimen, contributing to the limited clinical evidence in this area.

## Case 1

An 84-year-old Italian man affected by Hairy Cell Leukaemia, autoimmune anaemia, hypertensive cardiopathy, type 2 diabetes, and chronic renal insufficiency, was admitted to the haematological ward on October 9^th^, 2023, complaining of fever, abdominal pain, and lumbar pain for 1 month. Prior to hospitalization a total-body positron emission tomography (PET) had revealed increased glucose uptake within latero-cervical lymph nodes and L5 vertebra (Figure 1). A pulmonary nodule in the right upper lobe was documented with no increased glucose uptake. During hospitalization the patient underwent vertebral biopsy and lymph node aspirate, both showing chronic necrotizing granulomatous inflammation with negative microscopy and culture for *Mycobacterium tuberculosis* complex (MTC). Regardless, standard treatment for tuberculosis (TB) was started on November 16th (Figure 2A), and the patient was discharged in stable condition on December 29th.

**FIGURE 1:**
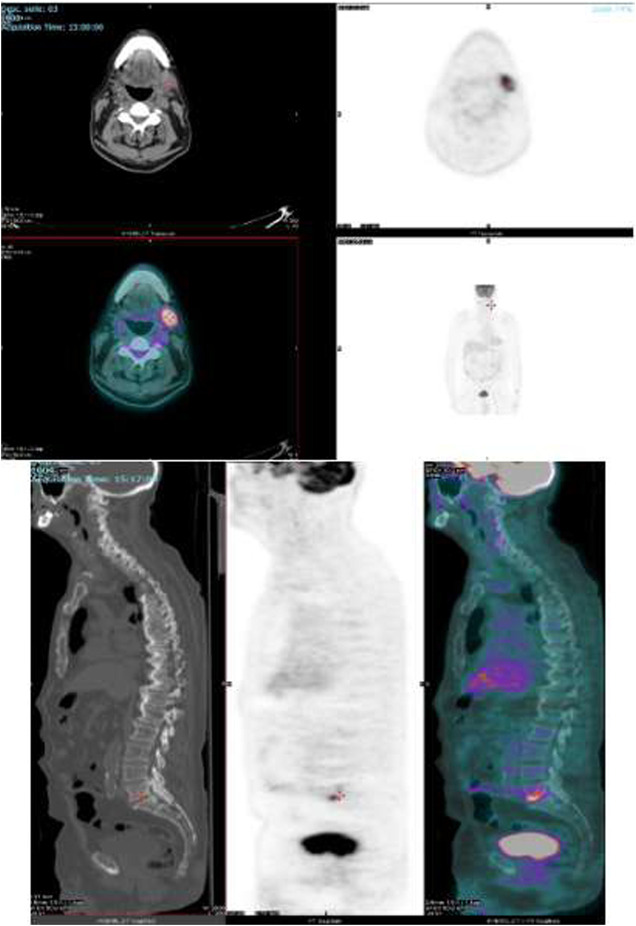
PET scan of Case 1 showing increased glucose uptake within latero-cervical lymph nodes and L5 vertebra.

**FIGURE 2:**
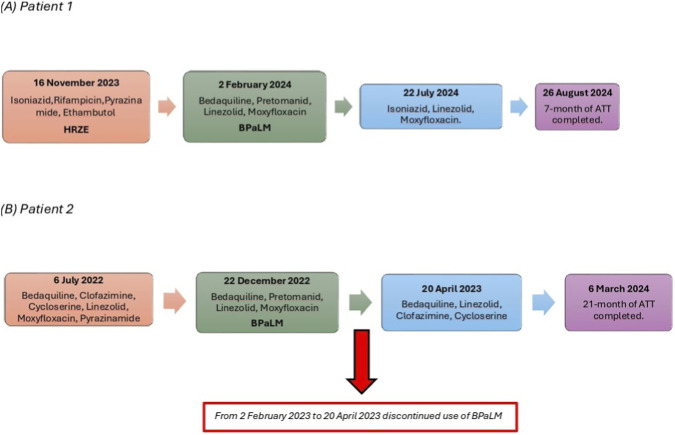
Anti-tubercular therapy (ATT) administered to Patient 1 **(A)** and 2 **(B)**. HRZE: Isoniazid, Rifampicin, Pyrazinamide, and Ethambutol. BPaLM: Bedaquiline, Pretomanid, Linezolid, and Moxifloxacin.

As no improvement in the conditions of the patient had occurred after 2 months of treatment, a Line Probe Assay (LIPA) test was applied to a deparaffinated sample of the bone biopsy, revealing the presence of *M. tuberculosis* genome along with a rpoB gene mutation conferring resistance to rifampicin, while sensitivity to isoniazid was retained. TB treatment was therefore switched to the BPaLM regimen on 2 February 2024. Bedaquiline and pretomanid were interrupted after 6 months in July, and treatment continued with linezolid, isoniazid, and moxifloxacin. By month seven of treatment significant side effects had emerged, including vision deterioration (chorio-retinal lesions and pathological ganglion cell layer (GCL) at the eye examination) and thrombocytopenia (PLT 48.000 cell/uL) and, on 26 August 2024, treatment was interrupted and considered completed. The eyesight slowly normalized, PLT count reverted to normal values (PLT 220.000 cell/uL), and no other signs of symptoms of disease emerged. In January 2025, 5 months after treatment completion, a magnetic resonance imaging (MRI) showed an unimproved radiological picture, and histological examination of a repeated vertebral biopsy showed focal reactive changes while *M. tuberculosis* search by direct, cultural, and molecular methods was negative. The histological picture was consistent with bone degeneration rather than TB reactivation.

## Case 2

A 38-year-old Moldavian man living in Italy since November 2021 with no remarkable past medical history, developed, in January 2022, skin proliferative lesions on the feet bilaterally, associated with persistent low-grade fever and weight loss. The right foot X-Ray showed calcification of the soft tissues and the MRI, performed on 5 May 2022, revealed osteomyelitis of the right tarsal cuneiform and scaphoid bones, while diffuse bone marrow alterations were observed in the midtarsal bone of the left foot. Several perineural masses were also present. In June 2022, he underwent orthopaedic surgery with removal of the perineural masses: the histological examination revealed chronic necrotizing granulomatous inflammation. No specimens were sent for microbiological examination.

On July 4th, he developed vertigo, confusion, and headache and presented to the Emergency Department of Mantua Hospital. Chest radiography revealed parenchymal thickenings, and he was admitted to the Infectious Diseases Department. A lumbar puncture was performed, showing hyperprotidorrachia and hypoglycorrachia at the cerebrospinal fluid (CSF) analysis, while GeneXpert tested positive for rifampicin-resistant *M. tuberculosis*. On July 6th, a six-drug antituberculosis regimen was started including bedaquiline, clofazimine, cycloserine, linezolid, pyrazinamide, and levofloxacin (Figure 2B), along with high-dose dexamethasone. Eventually MDR-TB was grown from the lungs and the bone, and a diagnosis of disseminated MDR-TB was made. In December 2022 (month 0) the second line drug sensitivity testing became finally available, and the initial regimen was switched to BPaLM, considering the excessive pill burden of the initial treatment and concerns regarding tolerability and adherence. On month 2 (February 2023), treatment was interrupted due to increased transaminases levels (AST 131 U/I, ALT 207 U/I) with dyspeptic symptoms. Twenty days later, after normalization of transaminases, the BPaLM regimen was successfully gradually re-introduced (first pretomanid, followed by linezolid, bedaquiline, and moxifloxacin). However, on month 4 (April 2023) treatment was stopped again due to hepatotoxicity. Two weeks later treatment was resumed with bedaquiline, linezolid, clofazimine and cycloserine. The cultural exam on the sputum tested negative in November 2023. The patient completed 21 months of treatment in March 2024. At 3-month follow up, the brain MRI was normal, and the patient was waiting for an orthopaedic intervention to correct the sequelae of TB on the feet.

## Discussion

Although comprehensive data on extrapulmonary RR/MDR-TB are limited, estimates suggest that CNS-RR/MDR-TB represents about 2% of all CNS-TB cases (Murthy, 2012), as well as bone RR/MDR-TB accounts for around 2% of all bone TB cases (Bonnet et al., 2022). Treatment outcomes vary significantly by site: mortality in CNS-MDR-TB reaches 30%–80% despite adequate therapy (Stadelman et al., 2020), whereas bone MDR-TB demonstrates better outcomes, with a French multicentre series of 23 patients reporting a ∼90% treatment success rate between 2007 and 2018 (Bonnet et al., 2022).

According to the 2022 WHO guidelines (World Health Organization, 2022) and 2025 updates (World Health Organization, 2025a) the BPaLM regimen is not recommended in CNS, bone, and miliary MDR-TB. These restrictions are due to pharmacokinetic and pharmacodynamic concerns regarding suboptimal drug penetration at these sites, particularly bedaquiline and pretomanid in the CNS (Upton et al., 2022). To date, only a single case report from the Nix-TB trial has documented the use of BPaL in CNS-XDR-TB (Howell et al., 2021), and no published data exist on its use in bone TB (Vanino et al., 2023).

We report two cases of MDR-TB of the bones or CNS (Table 1). Patient 1, affected by disseminated TB involving lungs, lymph nodes and bones, completed 7 months of therapy, six of which with the BPaLM regimen, and demonstrated no evidence of relapse at 1-year follow-up. Patient 2, presenting with disseminated TB involving lungs, bones, and CNS, received the BPaLM regimen that was discontinued after 4 months due to adverse events. In this patient, however, treatment interruption was attributable to adverse events rather than lack of efficacy.

**TABLE 1:** Clinical data on Patient 1 and 2. CNS: Central Nervous System; LIPA: Line Probe Assay; PCR: Polymerase Chain Reaction; BPaLM: Bedaquiline, Pretomanid, Linezolid, Moxifloxacin.

Clinical data	Patient 1	Patient 2
Age	84	38
Sex	Male	Male
Past Medical History	Hairy cell leukaemia Autoimmune anaemia Hypertensive cardiopathy Type 2 diabetes Chronic renal insufficiency	Removal of left foot Ganglion cysts
Site of Disease	Latero-cervical lymph nodes L5-S1 vertebra Lungs	Bones left foot CNS Lungs
Site of Diagnosis	L5-S1 vertebra (positive LIPA) Latero-cervical lymph nodes (positive LIPA)	CNS (PCR) Lungs (sputum smear) Bones (ganglion cysts histology)
Anti-tubercular therapy and duration	HRZE (*not effective*); BPaLM (*6 months*)	Bedaquiline, clofazimine, cycloserine, linezolid, levofloxacin (*6 months*); BPaLM (*4 months, unconsistently*); bedaquiline, linezolid, clofazimine and cycloserine (*11 months*)

The therapeutic management of patient one underscores two key issues: the feasibility of using BPaLM in disseminated/hard-to-treat TB and the optimal duration of therapy when applying short-regimens outside their pivotal trial populations. According to guidelines, patients with meningeal or bone tuberculosis should not receive less than 9 months of therapy (Nahid et al., 2016). Indeed, in the ZeNix and TB-PRACTECAL trials, selected centres extended BPaLM to 6–9 months in cases of extensive disease or delayed culture conversion. However, extending the treatment greatly increases the risk of adverse events from linezolid (such as peripheral neuropathy, myelosuppression, and optic toxicity), while the long-term safety of pretomanid is not well established.

According to the 2025 WHO guideline updates, another short therapeutic option for MDR-TB can be used, comprising bedaquiline, delamanid, linezolid, levofloxacin, and clofazimine (the BDLLfxC regimen) (World Health Organization, 2025a). Notably, BDLLfxC addresses therapeutic gaps in populations for whom BPaLM is not recommended, such as children, adolescents, and pregnant or breastfeeding women (PBFW) (World Health Organization, 2025a). However, this regime is not recommended for CNS, bone, and disseminated TB as well (World Health Organization, 2025a).

Our report contributes to the limited clinical evidence on the use of BPaLM in CNS and bone RR/MDR-TB. Shorter regimens such as BPaLM may therefore represent a viable alternative to prolonged therapies for extrapulmonary tuberculosis involving the CNS and bones. To revise the current WHO guidelines, which do not recommend BPaLM for tuberculosis involving the CNS and bones, further evidence is required. Given the diagnostic complexity and the rarity of these presentations, observational studies and case series are more likely to be published initially. However, if adequately encouraged and coordinated, they could help generate enough information to design clinical trials and eventually provide the evidence needed for a formal recommendation.

## Data Availability

The raw data supporting the conclusions of this article will be made available by the authors, without undue reservation.
